# Impending Doom: A Rare Case of Metastatic Myoepithelial Carcinoma

**DOI:** 10.7759/cureus.25785

**Published:** 2022-06-09

**Authors:** Emeka Ugwuegbulem, Swe Swe Hlaing, Gerson deFreitas, William McIntosh, Dhruvanshur Patel

**Affiliations:** 1 Internal Medicine, St. Luke's University Hospital, Bethlehem, USA; 2 Internal Medicine, Crozer-Chester Medical Center, Upland, USA

**Keywords:** hematogenous spread, sacral mass, deep soft tissue mass, myxoid sarcoma, myoepithelial carcinoma

## Abstract

Myoepithelial carcinoma is a rare malignant tumor arising from myoepithelial cells. The usual sites of occurrence are the oral cavity and pharynx with the majority of tumors arising from the salivary gland. However, there have been reported cases of myoepithelial carcinoma seen in the superficial soft tissue, upper respiratory tract, breast, skin, and GI tract. Deep soft tissue myoepithelial carcinoma is relatively uncommon.

Due to the rarity of this malignancy, consensus on appropriate therapy remains sparse. However, complete resection and/or adjuvant chemotherapy and radiotherapy have been advocated for non-metastatic localized diseases. Sadly, the reported outcome in patients with metastatic disease remains dismal.

In this case, we report a 79-year-old male incidentally found to have a deep soft tissue mass in the sacral area with a coexistent left axillary lymphadenopathy with biopsy and immunohistochemistry confirmation of metastatic myoepithelial carcinoma. He had a rapid clinical deterioration with subsequent fatality.

## Introduction

Although salivary gland myoepithelial carcinoma has been described extensively in the literature, deep soft tissue phenotypes have been sparsely described. Myoepithelial carcinoma is a group of rare malignant myoepithelial tumors that exhibit dual epithelial and smooth muscle phenotypes [1-10]. Soft tissue myoepithelial carcinoma has a male gender predominance with a wide age range distribution. The most frequent soft tissue sites are the extremities and limb girdles. The average tumor size was 8.7 cm, though it ranges from 2.0 cm to 21.6 cm [3-5].

Microscopically, these groups of tumors exhibit a wide range of morphological heterogeneity, necessitating the need for immunohistochemical analysis for accurate diagnosis. Furthermore, the accurate diagnosis of these forms of tumors is particularly important considering the high mortality related to tumor metastasis. 

Given the rarity of this malignancy, the clinical course and treatment modalities remain undefined. Multiple treatment modalities have been described in case reports, ranging from complete surgical resection with clear margins to chemotherapy and/or neoadjuvant /adjuvant radiotherapy [3-16].There are limited reports of deep soft tissue myoepithelial carcinoma with concurrent metastasis and rapid mortality [10-13].Therefore, we report a case of a 79-year-old male, with incidentally identified deep soft tissue mass in the sacral area and co-existent left axillary lymphadenopathy, which was biopsy confirmed to be a case of metastatic myoepithelial carcinoma.

## Case presentation

A 79-year-old male presented to the emergency room with a one-week duration of generalized weakness and a recent mechanical fall at home. His past medical history included type 2 diabetes mellitus, atrial fibrillation, hypothyroidism, coronary artery disease, and ischemic cardiomyopathy. Physical examination revealed normal vital signs and left axillary adenopathy.

**Figure 1:**
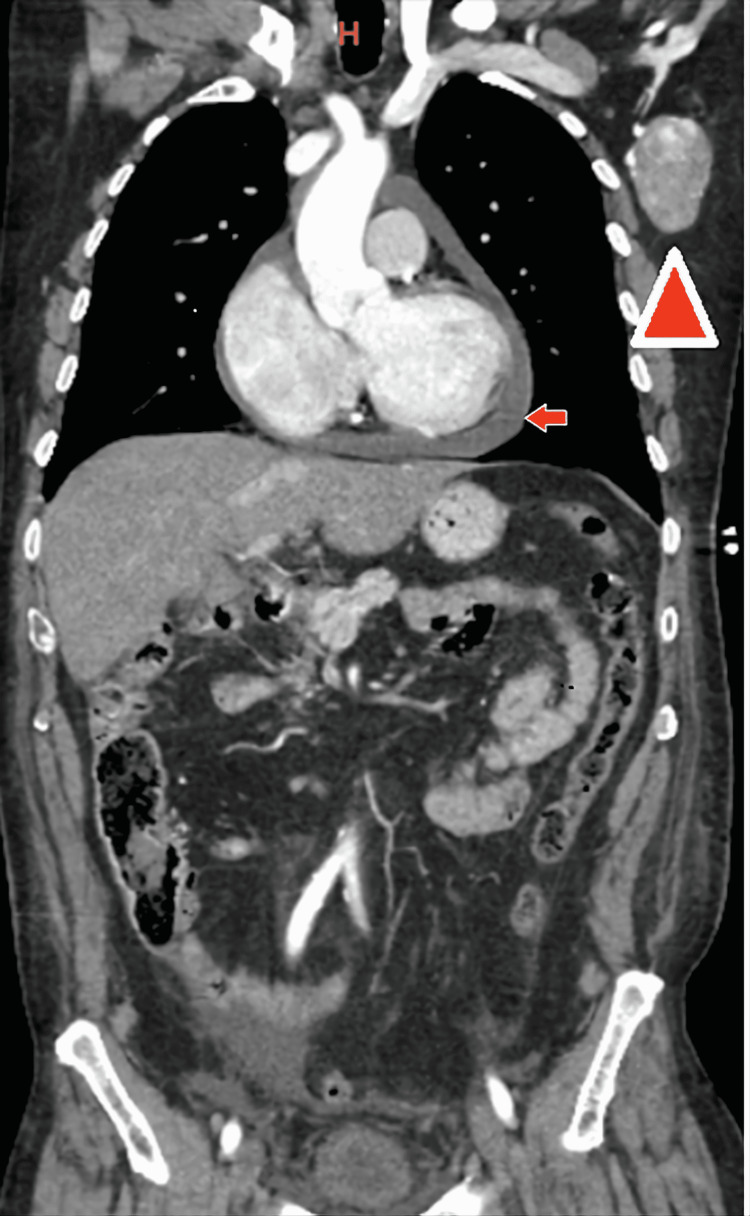
CT of chest, abdomen, and pelvis (coronal view) revealing large left lymphadenopathy (arrowhead) and pericardial effusion (small arrow)

**Figure 2:**
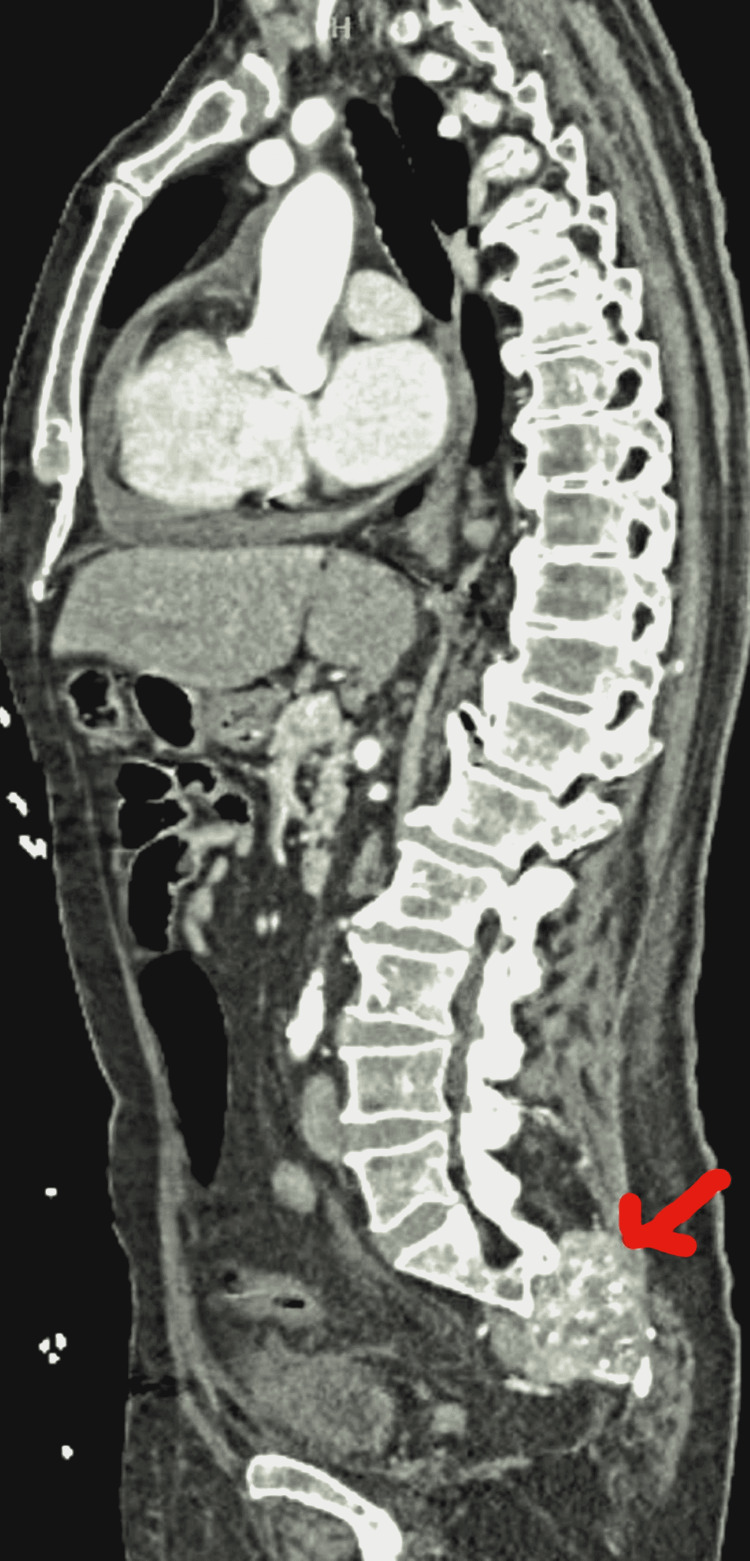
CT of chest, abdomen, and pelvis (sagittal view) revealing infiltrating sacral mass (red arrow)

**Figure 3:**
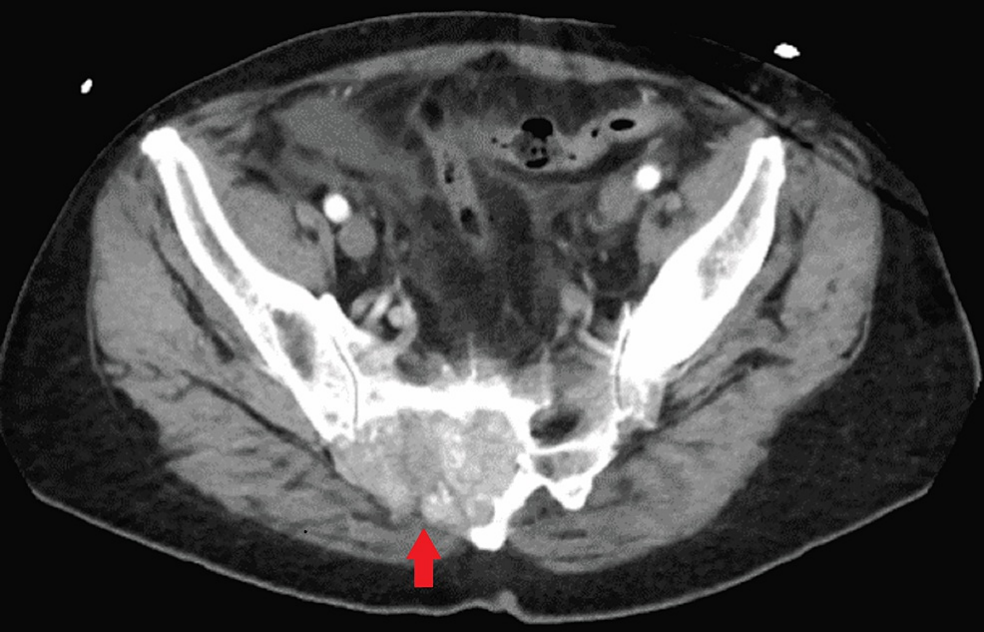
Pelvic CT (transverse view) revealing right sacral deep-seated mass

Complete metabolic panel (CMP) and complete blood count (CBC) were normal. Imaging for traumatic evaluation was performed. A computed tomography (CT) of the head was unremarkable. However, CT of the chest, abdomen, and pelvis revealed a moderately-sized pericardial effusion (Figure 1, arrowhead), a 7.1 cm x 6.9 cm x 4.7 cm heterogenous expansile soft tissue mass extending from the midline to the right sacroiliac joint (Figures 2, 3), and a 4.5 cm x 3.1 cm x 5.1 cm hypervascular enlarged left axillary lymph node (Figure 1).

Following CT-guided biopsy of the sacral mass, histology revealed a round to ovoid neoplasm, growing in a fine reticular pattern with fine chromatin in a background chondromyxoid stroma. Immunohistochemistry was positive for SOX- 10, cytokeratin AE1/AE3 (CKAE 1/3), CAM 5.2, B-cell lymphoma 2 (BCL-2), vimentin, and S100. However, it was negative for smooth muscle actin (SMA), p40, p63, epithelial membrane antigen (EMA), collagen IV, brachyury, synaptophysin, human melanoma black 45 (HMB-45), CD45, desmin, CD34, signal transducer and activator of transcription 6 (STAT6), and Melan-A. Integrase interactor 1 (INI 1) showed retained nuclear expression. A proliferation index (Ki-67) < 5%. No fusion transcript was detected by fluorescence in situ hybridization (FISH).

A core biopsy of the left axillary lymphadenopathy was obtained. The histologic section showed a cellular neoplasm composed of epithelioid cells with abundant pale eosinophilic cytoplasm growing in a mucinous /myxoid background stroma, with bland and inconspicuous nuclei. Immunohistochemistry was positive for SOX-10, pan-cytokeratin, and S100 while negative for CD117. Findings were described as morphologically and immunophenotypically identical to the sacral mass with no identifiable fusion transcript.

A transthoracic echocardiogram (TTE) of the heart showed a moderately-sized pericardial effusion with no tamponade physiology with the plan to manage conservatively. Subsequently, he was worked up for radiation therapy as an outpatient in conjunction with referral to a tertiary center for participation in a clinical trial.

Two weeks after initial hospitalization, he was admitted to the hospital with right lower extremity paresthesia and weakness. He then developed a neurogenic bladder and seizures. At this time, he proceeded with hospice care and declined further investigations. He died five weeks after his initial diagnosis.

## Discussion

Soft tissue myoepithelial carcinoma is an extremely rare form of soft tissue malignancy. In a large retrospective trial described by Hornick et al., which analyzed 101 cases of soft tissue myoepithelial cancers, there was a wide age variation with the mean age of presentation being 38 years and a male gender predisposition [1-6]. Across all age ranges, the most frequent sites of soft tissue myoepithelial carcinoma were the upper and lower extremities. However, there was a reported case of gluteal involvement with sacral extension [2-12].

These tumors seem to share morphologic, immunophenotypic, and genetic features with their salivary gland equivalents [7].Histologically, myoepithelial tumors show a wide spectrum of cellular and architectural morphologies. Most tumors are mixed with a clear, spindle, epithelioid, and plasmacytoid cells forming nests, cords, or solid sheets in chondromyxoid or collagenous/hyalinized stroma with a cytoplasm that tends to be clear or eosinophilic. In addition, there is a variable expression of cytoplasmic filaments and muscle markers [3,4].

Given the varied histologic presentation of this group of neoplasm, immunohistochemistry plays a huge role in appropriate confirmatory identification. Overall, most of the antibodies used to detect myoepithelial neoplasm in immunohistochemistry target myofilament and keratin [6]. It is advised that myoepithelial neoplasms are best examined with a tumor panel that includes all antibodies to broad-spectrum keratins, high-molecular-weight keratins, and myofilaments [6]. About 95% of tumors express reactivity to keratin (CKAE 1/3), S100, EMA, PAN-K, CAM 5.2, pan-cytokeratin, calponin, SMA, muscle-specific actin (MSA), smooth muscle myosin, P63 protein, glial fibrillary acidic protein (GFAP), CD10, desmin, and p63 [2-17]. In our case, the tumor was immunoreactive to SOX- 10, CKAE 1/3, CAM 5.2, BCL-2, vimentin, and S100 confirming the diagnosis of myoepithelial carcinoma. Additionally, staining for INI-1 can be helpful. About 10-20% of myoepithelial carcinomas demonstrate loss of INI-1 expression, which is due to chromosome 22q deletion. However, the clinicopathologic relevance of Ewing sarcoma breakpoint region (EWSR 1) gene rearrangement remains undefined. It has been shown to be relatively frequent in certain subsets of deep soft tissue tumors and can aid in serving as a relevant diagnostic tool in tricky cases [18]. Currently, there are conflicting reports regarding its prognostic significance. Nevertheless, an aggressive case with EWSR1-negative gene rearrangement was reported by Aparicio et al. [19], which is consistent with our case as no fusion transcript was detected on FISH.

At present, there is no consensus evidence-based treatment modality for this neoplasm particularly due to its rarity. However, in a large single-center retrospective study described by Chamberlain et al., the recommendation for primary treatment of localized disease was complete surgical resection with or without radiation therapy [14]. Neoadjuvant radiation therapy to debulk the tumor can be considered in certain groups of patients at risk of significant tissue/limb loss. In a case report by Kabarriti et al., neoadjuvant radiotherapy was followed by wide excision surgery and post-surgical brachytherapy. The resected specimen revealed only 10% viable tumor cells indicating excellent tumor response. This further reinforces the benefit of radiotherapy as a treatment modality [16]. Platinum-based chemotherapy has been utilized in recurrent or metastatic disease following resection with limited benefits [6]. Despite complete surgical resection, there is about a 43% rate of local recurrence [3] and a 40-50% rate of metastasis [9]. The use of combination chemotherapy to treat Ewing sarcoma (vincristine, doxorubicin, and cyclophosphamide alternating with ifosfamide and etoposide was seen to produce a dramatic response in EWSR-positive tumors [20]. Unfortunately, this has not significantly been replicated in most cases of EWSR-positive myoepithelial carcinoma. Despite the use of chemotherapy, the median progression-free survival for first-line chemotherapy was reported as 9.3 months. It is suggested that this could be more so due to significant uncertainty in the clinical behavior of this malignancy [14].

Metastatic lesions have been described in the brain, spinal canal, cecum, and lungs. Given this pattern of metastasis, a hematologic route of metastases is reasonable. This most likely explains the noncontiguous seeding to the right axillary lymph node as seen in our patient. Prognosis with metastasis remains grim given limited therapeutic options. Therefore, this highlights the need to encourage participation in clinical trials, although sparsely available, in highly specialized multidisciplinary units to establish consensus treatment guidelines and improve patient outcomes [17].

## Conclusions

This case presentation is unique given the divergent sites of metastasis, which support a hematogenous route of metastasis. In addition, it highlights the high mortality associated with metastatic myoepithelial carcinoma as evidenced by the rapid progression of the disease with subsequent deterioration in the patient's quality of life and performance status. There is an immense need to obtain more understanding of this disease to establish consensus management guidelines and improve outcomes. Given the scarcity of data regarding the clinical course and management, clinicians should strongly consider referral to specialist centers for participation in clinical trials with a multidisciplinary team care advocated.
